# Some Observations Respecting the Nature and Treatment of Venereal Diseases

**Published:** 1821-03

**Authors:** Leonard Gillespie

**Affiliations:** lately Physician to the Fleet, &c.


*Some Observations respecting the Nature and Treatment of Venereal Diseases.* By LEONARD GILLESPIE, M.D. lately Physician to the Fleet, &c.*

THE dangerous, and sometimes fatal, consequences resulting from long-continued courses of mercurial medicines, in patients affected with complaints arising originally from venereal infection, but protracted from a scorbutic, scrofulous, herpetic, or other bad habit of body, strengthened by intemperance, and often aggravated by the supposed specific, occur too often in private practice, and frequently present themselves in naval hospitals. Some instances of this sort occurred during the course of this year (1798), in which a gangrenous state of the penis or of the inguina, after suppurated buboes, happening in persons on a sea-diet and of a scorbutic habit, appeared to be induced by a too-long continued use of mercurials. It is much to be lamented that the minds of many young practitioners, and those of patients labouring under the diseases termed venereal, are so strongly impressed with the absolute necessity of administering mercurials in these complaints, as long as any symptoms whatever appear, that common sense is often disregarded by them: thus it is sufficiently obvious that, when mercury, administered in an ulcerated state of the genitals, occasions an aggravation of the symptoms, this metal, in

* It seems right that the following remarks, contained in a letter from Dr. Gillespie to Dr. Hutchinson, should accompany this paper. The propriety of adducing them here appears, indeed, to be so obvious, that we have not thought it necessary to delay the insertion of the paper whilst we consulted Dr. Gillespie respecting his intentions in regard to the publication of them.—EDIT.

"Retired for several years from practice, uninfluenced by any hope of acquiring fame or fortune from novel or popular opinions, and free from any dread of being regarded as a heretic in medicinal doctrines, I have beheld, for some years past, with much interest, the various publications and discussions which have taken place relative to the specific nature of syphilis, and the use and abuse of mercury in that disease.

"This induces me to address to you, herewith, a literal extract from my journal of diseases, observed at the naval hospital of Fort Royal, Martinique, in the year 1798, written by me there in 1799.

"Attentive as I have since been to the subject of this important investigation, I have not seen or read any thing which does not tend to confirm me in the opinions which I have advanced in the above-mentioned extract, and which the experience and writings of so many practitioners seem of late to strengthen.

"After a lapse of more than twenty years since the journal mentioned was written, I may hope that, in offering you this extract for publication in your esteemed Journal, you, as well as the public at large, will absolve me from the imputation of precipitancy in obtruding a doctrine, which, though looked on at present as an innovation in medicine, has, perhaps, had at all times supporters, as may be traced by consulting the immense number of authors who have treated of this disease, from Moses to our own times; and that you will believe, the duty incumbent upon every man to endeavour to alleviate the sufferings of his fellow-creatures, is the sole inducement which influences me in requesting you to give this paper a place in your Journal."

the room of being looked on as a specific for this complaint, should, on the contrary, be rather regarded as an anti-specific in the individual in question.

These impressions doubtless arise, 1st, from the supposed specific nature and identity of *venereal virus,* as it is usually termed, in consequence of which it is assimilated with diseases which have a regular and almost invariable procession in their phenomena, periods, and termination,—as plague, small-pox, measles, &c. and the generation of which in the individual would seem to be a very rare occurrence; whereas, venereal affections, originating in all periods and nations where impure and promiscuous venery is practised, in the great majority of cases which present themselves, only exhibits a slight topical affection, often curable by the simple efforts of nature, and rarely producing a general affection of the system. This must be allowed to be the most general appearance of the disease; for, the ravages which it made in Europe, in the latter part of the fifteenth and sixteenth centuries, amongst the scorbutic seamen of Columbus, the licentious French soldiery in the kingdom of Naples, and, finally, throughout a great part of Europe, can only be accounted for, as occasioned by an epidemic of a particular nature, arising from the constitution of the air and preceding seasons, spread by contagion; which seems to be the invariable opinion of the first authors who wrote on that epidemic.

In such rare instances as at this day occur of a general contamination of the habit after venereal infection, producing the two hundred and thirty-four symptoms of the disease, said to be enumerated by the Italian Professor Brassavolino,* and since greatly increased by his successors, the cause would seem to reside in the habit of body of the patient, whether affected by the inflammatory, scorbutic, scrofulous, ulcerative, arthritic, leprous, or other diathesis, which thus gives rise to the anomalous and irregular class of symptoms which have been termed constitutional syphilis.

In such cases, the reverse takes place to what happens in diseases which have really a specific nature, when the number of those exposed to the contagion without essaying the regular attack of the disease is less remarkable, compared to that of those who suffer the usual symptoms. In syphilis, on the contrary, the constitutional symptoms do not occur, perhaps, once

* This author wrote in 1551, and has a place in the Aphrosodiacus of Boerhaave. He was such an admirer of mercurial inunction in the cure of lues venerea, that he says that "it would seem rather to have been found out by the gods than by men." The learned Scaliger seems to write justly of Brassavolino, in saying—

"Nihil alind eum virum fuisse, quam cymbalum ineptæ medicorum plebis."

in fifty patients exposed to the infection, and seem (speaking from experience) often induced by a preposterous mode of treatment.

The impression so generally fixed on the mind of young practitioners, and many venereal patients, that mercury alone is the grand specific, remains to be discussed.

To form a table of the calamities which the abuse of mercury in the venereal disease has occasioned since the empirical introduction of it into practice, would require a large volume. To those who have not sufficient evidence of this abuse, an examination of the collection of authors, published by Boerhaave, on Lues Venerea, termed Aphrosodiacus, will, it is presumed, remove all doubts on that head. Yet it may be alleged, that the abuse of mercury, or any other remedy, should not militate against the appropriate administration of it; which axiom cannot be disputed. But, when we behold the debilitated victims to syphilis, or, more properly, to the abuse of the supposed specific, with which our hospitals and our streets abound, and compare their sufferings with the easy and safe cure for the same disease which the American and African natives find in the efforts of nature to relieve herself, in regimen, and in their own vernacular remedies, shall we not be inclined to judge that, in this disease, medicine, as practised according to the rules of art, does more harm than good, and that the remedy is often worse than the disease?

This digression may perhaps be deemed misplaced, and the subject of it would more properly demand a more copious investigation: it is here brought forward principally in behalf of young naval and military surgeons, who are earnestly recommended to be cautious in the administration of mercurials to seamen and soldiers in venereal complaints, and to reflect,

First, That the best authors allow, and daily experience proves, that the simple topical affections of gonorrhœa, inflammation and ulceration of the penis, scrotum, and inguinal glands, are curable without any administration of mercury; which often aggravates both the inflammatory and the ulcerative stages of these complaints, though sometimes useful in the latter as an escharotic and detergent, in the form of corrosive sublimate, &c. externally.

Secondly, that in scorbutic seamen and soldiers, so much exposed, from regimen and manner of life, to scurvy, itch, herpes, ulcers, and arthritic affections, these complaints, whether simple or variously complicated, are often mistaken, both by the patient and surgeon, for syphilis, and maltreated, in consequence, by long and debilitating courses of mercurials; mankind being unfortunately too prone to attribute to occult causes maladies arising from errors in regimen. In one of the cases of supposed lues which occurred this year, the patient, during a long passage, had been put on a course of mercurials on account of a chancre, which this treatment had aggravated into a gangrenous, sloughy sore, destroying a considerable part of the glans penis. The patient was extremely irritable, restless, and emaciated. A change of diet; the free use of bark, and sarsaparilla in powder and decoction; the occasional administration of opiates internally; whilst the ulcer was deterged with the expressed juice of worm-grass, or choenopodium anthelminticum, and dressed with a pledget of mel Ægyptiacum and ointment of storax; brought the sore to the state of a clean ulcer, tending to cicatrization. In another more acute case of gangrene of the penis, the best effects were experienced from a poultice of the pulp of roasted limes, applied over a pledget of ointment of storax, &c.

*Paris; Rue Neuve Saint-Augustin, No.* 41.
*Jan.* 15, 1821.

